# FANCM suppresses DNA replication stress at ALT telomeres by disrupting TERRA R-loops

**DOI:** 10.1038/s41598-019-55537-5

**Published:** 2019-12-13

**Authors:** Xiaolei Pan, Yun Chen, Beena Biju, Naveed Ahmed, Joyce Kong, Marti Goldenberg, Judy Huang, Nandakumar Mohan, Stephanie Klosek, Kian Parsa, Chia-Yu Guh, Robert Lu, Hilda A. Pickett, Hsueh-Ping Chu, Dong Zhang

**Affiliations:** 1 Department of Biomedical Sciences, College of Osteopathic Medicine, New York Institute of Technology, Old Westbury, NY 11568 USA; 2 Institute of Molecular and Cellular Biology, National Taiwan University, Taipei, Taiwan; 3 Telomere Length Regulation Unit, Children’s Medical Research Institute, University of Sydney, Westmead, New South Wales Australia

**Keywords:** Telomeres, Genetics research

## Abstract

Cancer cells maintain their telomeres by either re-activating telomerase or adopting the homologous recombination (HR)-based Alternative Lengthening of Telomere (ALT) pathway. Among the many prominent features of ALT cells, C-circles (CC) formation is considered to be the most specific and quantifiable biomarker of ALT. However, the molecular mechanism behind the initiation and maintenance of CC formation in ALT cells is still largely unknown. We reported previously that depletion of the FANCM complex (FANCM-FAAP24-MHF1&2) in ALT cells induced pronounced replication stress, which primarily takes place at their telomeres. Here, we characterized the changes in ALT associated phenotypes in cells deficient of the FANCM complex. We found that depletion of FAAP24 or FANCM, but not MHF1&2, induces a dramatic increase of CC formation. Most importantly, we identified multiple DNA damage response (DDR) and DNA repair pathways that stimulate the dramatic increase of CC formation in FANCM deficient cells, including the dissolvase complex (BLM-TOP3A-RMI1/2, or BTR), DNA damage checkpoint kinases (ATR and Chk1), HR proteins (BRCA2, PALB2, and Rad51), as well as proteins involved in Break-Induced Replication (BIR) (POLD1 and POLD3). In addition, FANCD2, another Fanconi Anemia (FA) protein, is also required for CC formation, likely through promoting the recruitment of BLM to the replication stressed ALT telomeres. Finally, we demonstrated that TERRA R-loops accumulate at telomeres in FANCM deficient ALT cells and downregulation of which attenuates the ALT-associated PML bodies (APBs), replication stress and CC formation. Taken together, our data suggest that FANCM prevents replisomes from stalling/collapsing at ALT telomeres by disrupting TERRA R-loops.

## Introduction

The fidelity of DNA replication may be one of the most crucial factors affecting cancer incidence. Based on cancer genome sequencing and epidemiology studies, Tomasetti and colleagues proposed that the majority of cancers may arise from random mutations generated during routine DNA replication in normal stem cells^1,2^.

Certain regions of human genome pose more challenges to the DNA replication machinery, i.e. the replisome, than others, for example rDNA loci, centromeres, common fragile sites (CFS), subtelomeres and telomeres. Because of unique sequences, organization, and chromatin status at telomeres/subtelomeres, replisomes are especially prone to pausing/stalling at these regions. Mammalian telomeres consist of G-rich tandem hexanucleotide repeats, the large majority of which contain the sequence, TTAGGG. A particular DNA secondary structure, called G-quadruplex or G4, is prone to forming at telomeres^3,4^. A long non-coding RNA (lncRNA) transcribed from the telomere/subtelomere regions, TERRA, could base-pair with telomeric DNA and form a DNA:RNA hybrid, i.e., an R-loop^5,6^. The telomeric G-rich overhang folds back, invades the double-stranded region of telomeres, and forms the telomeric loop (T-loop) and the displacement loop (D-loop)^7,8^, which are facilitated and stabilized by a multi-subunit protein complex called Shelterin^9,10^. All of these DNA-DNA, DNA-RNA, and DNA-protein structures need to be overcome in order for replisomes to successfully replicate telomeres during each cell cycle. Failure to do so leads to replication stress at telomeres.

Being able to maintain telomere length is especially important for the long-term survival of cancer cells. 85–90% of cancers achieve this by re-activating their telomerase (TEL+), a ribonucleoprotein complex that extends telomeres via its reverse transcriptase activity^11^. 10–15% of cancers adopt the Alternative Lengthening of Telomere (ALT) pathway^12^. Recently, in a small group of aggressive cancers, neither telomerase activity nor ALT was detected, and yet they can still survive for hundreds of generations, suggesting that certain cancers may be able to survive in the absence of the two known telomere maintenance mechanisms (TMM)^13^. The biochemical and biological functions of telomerase are well-established^10^. In contrast, the detailed mechanisms of how ALT is initiated and maintained are less well defined and warrants further investigation^14,15^. ALT cells manifest certain shared characteristics, including increased frequency of telomere sister chromatin exchange (tSCE), increased formation of ALT-associated PML bodies (APBs), telomere dysfunction-induced foci (TIFs), and the appearance of large amounts of extrachromosomal telomeric repeats (ECTR), such as C-circles (CC) and G-circles (GC)^14^. In addition, ALT telomeres are more heterogeneous in length and some of them can be quite long compared to those in TEL+ cells^12^. Among the features that help to distinguish ALT from TEL+, CC formation is shown to be the most robust, specific, and quantifiable biomarker for ALT^16,17^.

In addition to factors involved in Homologues Recombination (HR), recent studies indicate that a unique DNA replication/repair pathway, called Break Induced Replication (BIR), plays an important role in ALT pathway^15,18–22^. In lower organisms, there also exists a similar pathway to render the survival of the organism in the absence of a functional telomerase. For example, in budding yeast, *Saccharomyces cerevisiae* (*S. cerevisiae*), inactivation of telomerase induces the appearance of two types of survivors: Type I and Type II, both of which also utilize the HR-mediated processes to maintain their telomeres and avoid telomere shortening induced cell death^23^. BIR is also important for both types of survivors^24^. Intriguingly, over-expression of the *S. cerevisiae MPH1* gene, the yeast homolog of human FANCM, strongly suppresses the BIR at certain double-stranded breaks (DSBs)^25^.

Human *FANCM* belongs to a family of genes that are highly conserved^26,27^. Its orthologs have been identified in many organisms, ranging from prokaryote - archaeal *Pyrococcus furiosus*, to rodent – *Mus musculus*. All members of the FANCM family of proteins contain seven conserved motifs that are found in the superfamily 2 (SF2) helicases, including a DEAH box (motif II) that is important for ATP hydrolysis^28^. However, unlike other members of the FANCM family of proteins, there is no significant unwinding activity detected towards various double-stranded DNA structures by the human FANCM. Instead, it acts on branched DNA structures that mimic stalled replication forks or Holiday junction intermediates and promotes fork branch migration, suggesting that FANCM likely plays an important role in repairing/re-starting a stalled/collapsed replication fork^29^. Recently, Schwab and colleagues showed that recombinant human FANCM is also able to resolve a generic R-loop via its branch migration activity *in vitro*^30^. They further showed that depletion of FANCM induced the upregulation of R-loops as detected by immunofluorescent staining using the S9.6 monoclonal antibody, which detects the DNA:RNA hybrid^31^. However, where those R-loops located are unclear. FANCM is also a major player in repairing inter-strand crosslinking (ICL) DNA lesions^32^. During ICL repair, FANCM, along with its three binding partners, FAAP24, MHF1, and MHF2, helps to recruit the FA core complex to ICLs and promote monoubiquitination of FANCD2^33–38^.

Recently, we discovered that depletion of FANCM in multiple ALT cell lines induces a pronounced replication stress response, which primarily takes place at telomeres^39^. We showed that many DNA damage response (DDR) factors, including BLM, BRCA1, ATR, Chk1, and Rad51 facilitate this endogenous replication stress response. Building on these studies, we demonstrated here that depletion of FANCM also induces a dramatic increase of two key features of ALT: APBs and CC formation. Using CC formation as the readout, we performed a targeted screening and identified many DDR factors that affect the upregulation of CC in FANCM deficient ALT cells. Finally, we showed that depletion of FANCM leads to a pronounced accumulation of TERRA R-loops at the ALT telomeres. Taken together, we propose that in ALT cells, FANCM actively disrupts the TERRA R-loops during telomere replication thus suppressing the replication stress at telomeres.

## Results

### Depletion of FANCM in ALT cells leads to a dramatic increase of APBs and C-circles

**Figure 1:**
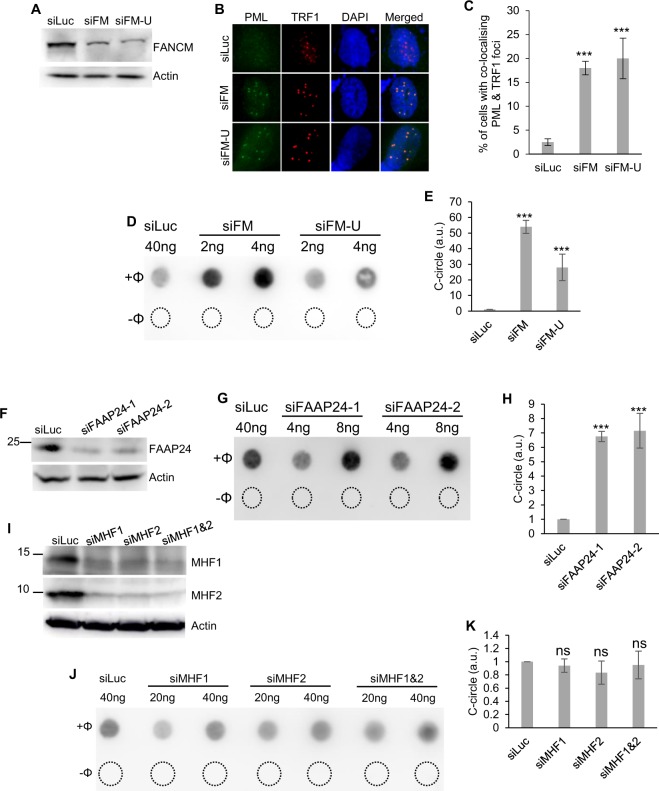
Depletion of FANCM and FAAP24, but not MHF1 and MHF2, stimulates dramatic C-circles formation in ALT cells. (**A,F,I**) Efficient depletion of the FANCM complex using siRNA. (**B,C**) siRNA transfected U2-OS cells were co-stained with antibodies recognizing PML and TRF1. (**D,E,G,H,J,K**) siRNA transfected U2-OS cells were used for C-circle analysis. “−Φ” indicates samples with no Phi(Φ)29 DNA polymerase added. The amount of C-circles in siLuc transfected cells is used to normalize C-circles in other siRNA transfected cells. All error bars are standard deviation of the mean obtained from three different experiments. Standard two-tailed Student’s t-test: *p < 0.05, **p < 0.01, ***p < 0.001, ns – not significant.

In a recent study, we demonstrated that depletion of FANCM and three of its binding partners, FAAP24, MHF1, and MHF2, in ALT cells induced different severity of replication stress at their telomeres^39^. As mentioned above, ALT cells often manifest certain molecular and cellular features. We tested whether depletion of FANCM affects some of the ALT properties, such as the formation of APBs and CC. As we showed previously^39^, transfection of U2-OS cells with two different siRNA targeting FANCM, siFM and siFM-U, efficiently depleted cellular FANCM (Fig. 1A). siFM targets the coding region of FANCM mRNA, while siFM-U targets the 3′ untranslated region (3′ UTR) of the FANCM mRNA. As seen in Fig. 1B–E, depletion of FANCM induced a dramatic increase of both APBs (~20X) and CC (~20X–50X) in U2-OS cells. When FANCM was depleted in another ALT cell line, Saos-2, an approximately 5-fold increase of CC formation was observed (Fig. S1). Because the basal level of CC in Saos-2 is approximately 3- to 4-fold higher than in U-2 OS^16^, it is not surprising that depletion of FANCM in Saos-2 induced a milder increase than in U-2 OS. In stark contrast, depletion of FANCM in two TEL + cell lines, HeLa and MG63, did not induce any increase of CC formation (Fig. S1), suggesting that FANCM deficiency induced CC formation is unique to ALT cells.

As mentioned above, FAAP24, MHF1, and MHF2 are often found in complex with FANCM. Next, we tested whether depletion of FAAP24, MHF1, and MHF2, can also induce CC formation. As shown in Fig. 1F–H, depletion of FAAP24 using two different siRNAs induced an approximately 7-fold increase of CC formation. To our surprise, depletion of MHF1, or MHF2, or MHF1 and MHF2 in combination, has little effect on CC formation (Fig. 1I–K). Intriguingly, we did not observe pronounced telomere length changes in FANCM, or FAAP24, or MHF1, or MHF2, transiently depleted cells (Fig. S2).

Taken together, our data indicate that FANCM likely functions together with FAAP24, but not MHF1 and MHF2, to inhibit the formation of CC in ALT cells.

### The dissolvase complex plays an important role in the checkpoint activation, single stranded DNA (ssDNA) formation, and CC formation in FANCM deficient ALT cells

**Figure 2:**
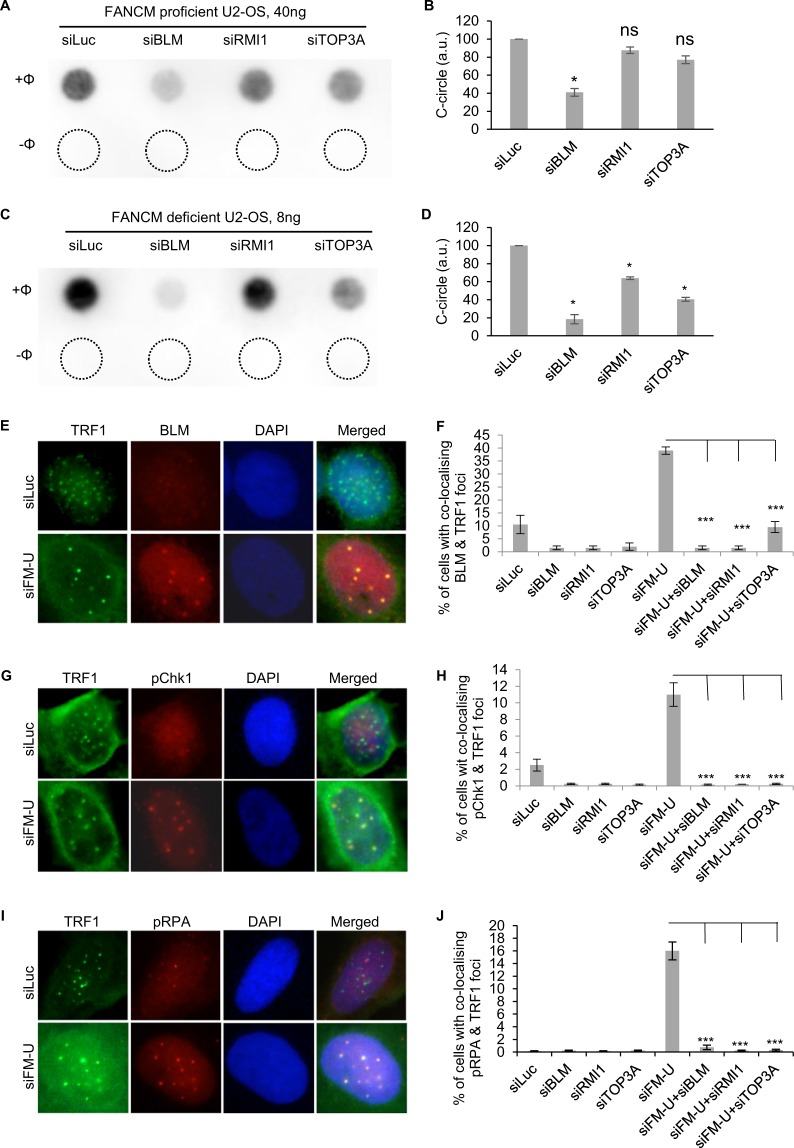
The dissolvase complex stimulates C-circles formation and promotes checkpoint activation and ssDNA formation in FANCM deficient ALT cells. (**A–D**) U-2 OS cells were transfected with siRNA targeting luciferase (siLuc), BLM, RMI1, TOP3A, together with either FANCM siRNA (siFM-U) (labelled as FANCM deficient U-2 OS) or with siLuc (labelled as FANCM proficient U-2 OS). Genomic DNA was extracted and used for C-circle assay. “−Φ” indicates samples with no Phi(Φ)29 DNA polymerase added. The amount of C-circles in siLuc transfected cells is used to normalize C-circles in other siRNA transfected cells. (**E–J**) siRNA transfected U2-OS cells were co-stained with antibodies recognizing BLM and TRF1, or pChk1 and TRF1, or pRPA and TRF1. All error bars are standard deviation of the mean obtained from three different experiments. Standard two-tailed Student’s t-test: *p < 0.05, **p < 0.01, ***p < 0.001, ns – not significant.

Our recent study showed that FANCM, BLM, and BRCA1 collaboratively facilitate the replication stress at ALT telomeres^39^. Next, we investigated whether BLM and its two binding partners - RMI1 and TOP3A, which are part of the dissolvase complex, are involved in regulating the CC induction in FANCM depleted cells. As seen in Fig. 2A,B, only the depletion of BLM, but not RMI1 and TOP3A, moderately inhibited the CC formation in FANCM proficient ALT cells (siLuc co-transfected cells). In contrast, depletion of all three proteins attenuated the robust increase of CC formation in FANCM deficient cells (siFM-U co-transfected cells) (Fig. 2C,D), with the depletion of BLM showing the strongest effect. In our previous study, we found that BLM are actively recruited to the replication stressed telomeres in FANCM deficient ALT cells^39^. As seen in Fig. 2E,F, depletion of RMI1 or TOP3A dramatically decreased the telomeric BLM foci, indicating that the recruitment of BLM to replication stressed telomeres requires an intact dissolvase complex. Furthermore, all three proteins are also required for checkpoint activation and ssDNA formation at telomeres in FANCM deficient ALT cells (Fig. 2G–J).

### Multiple DNA damage response and DNA repair pathways are implicated in the upregulation of C-circles formation in FANCM deficient ALT cells, including DNA damage checkpoint, HR, and BIR

CC formation is the most specific and quantifiable biomarkers for ALT cells^16^. Therefore, proteins that affect CC formation likely also play an important role in the ALT pathway. Since we observed a dramatic upregulation of CC formation in FANCM depleted cells (Figs. 1D,E and S1), we selected 12 additional DDR genes and performed a targeted screening to identify genes that play a role in regulating the CC formation in either FANCM proficient cells or FANCM deficient cells. The siRNA knockdown efficiency for the genes tested is shown in Fig. S3. Results of the CC formation screening are summarized in Table 1. In addition to the dissolvase complex, we found that two important checkpoint kinases (ATR and Chk1), three HR proteins (BRCA2, PALB2, and Rad51), two proteins involved in BIR (PolD3 and PolD1), a translesion DNA polymerases, Polη, as well as a key Fanconi Anemia protein, FANCD2, also stimulate the CC formation in FANCM deficient U-2 OS. Depletion of BRCA1, Rad52, and two important DNA end resection nucleases (CtIP and Mre11) did not affect the CC formation in FANCM deficient cells. Depletion of the two cohesion-like proteins, Smc5 and Smc6, did not affect the CC formation either even though it was shown previously that they are implicated in the ALT pathway^40^. Interestingly, in FANCM proficient U-2 OS, depletion of many DDR genes leads to a mild increase of CC formation. We speculate that this could be due to mild telomere damage induced in those cells.

**Table 1:** Genes affecting C-circles formation in either FANCM proficient or FANCM deficient ALT cells. The targeted screening was done using the same CC assay as describe in the methods. The values shown are the mean obtained from at least two independent experiments. ± : standard deviation. Standard two-tailed Student’s t-test: *p < 0.05, ns – no significance.

	FANCM Proficient	FANCM Deficient
Control (Luc)	100	100
**Dissolvase**		
BLM	41 ± 4.2(*)	18.5 ± 4.9(*)
TOP3A	77 ± 4.2(ns)	41 ± 2.2(*)
RMI1	88 ± 3.5(ns)	64 ± 1.4(*)
**Checkpoint kinase**		
ATR	153 ± 4.9(*)	62 ± 2.1(*)
Chk1	145 ± 2.1(*)	17 ± 2.8(*)
**Break-induced replication**		
PolD3	88 ± 12(ns)	34 ± 5.7(*)
**DNA polymerase**		
PolD1	115 ± 7.1(ns)	35 ± 7.1(*)
PolH	102 ± 12(ns)	25.5 ± 7.8(*)
**Homologous recombination**		
BRCA1	110 ± 11.3(ns)	102 ± 15.6(ns)
BRCA2	237 ± 9.2(*)	53 ± 1.3(*)
PALB2	124 ± 4.9(ns)	62 ± 1.4(*)
Rad51	89 ± 2.1(ns)	46 ± 2.8(*)
Rad52	106 ± 14(ns)	98 ± 10.6(ns)
**DNA end resection**		
CtIP	92 ± 2.1(ns)	104 ± 11.3(ns)
Mre11	249 ± 12(*)	105 ± 21.9(ns)
**Cohesion-like complex**		
Smc5	194 ± 7.1(*)	87 ± 6(ns)
Smc6	264 ± 4.9(*)	88 ± 7.8(ns)
**Others**		
FANCD2-1	226 ± 8.5(*)	43 ± 4.2(*)
FANCD2-2	175 ± 7.1(*)	26 ± 5.7(*)

The different requirement for CC formation in FANCM proficient vs the FANCM deficient ALT cells is quite intriguing (Table 1). Since transient depletion of FANCM induces such a robust replication stress response at ALT telomeres within such a short period of time (within days), this potentially mimics the condition of the “initial” generation of CC when telomeres first encountered massive replication stress in the absence of telomerase. As for the FANCM proficient ALT cells, they have been in the “ALT state” for a long time (years or decades) and have likely developed certain strategy to cope with low level and persistent replication stress at their telomeres. Therefore, the CC detected in those cells likely reflects the “maintenance” stage of CC formation.

### BRCA2, PALB2, and FANCD2 promote the recruitment of BLM to the replication stressed telomeres in FANCM deficient ALT cells

**Figure 3:**
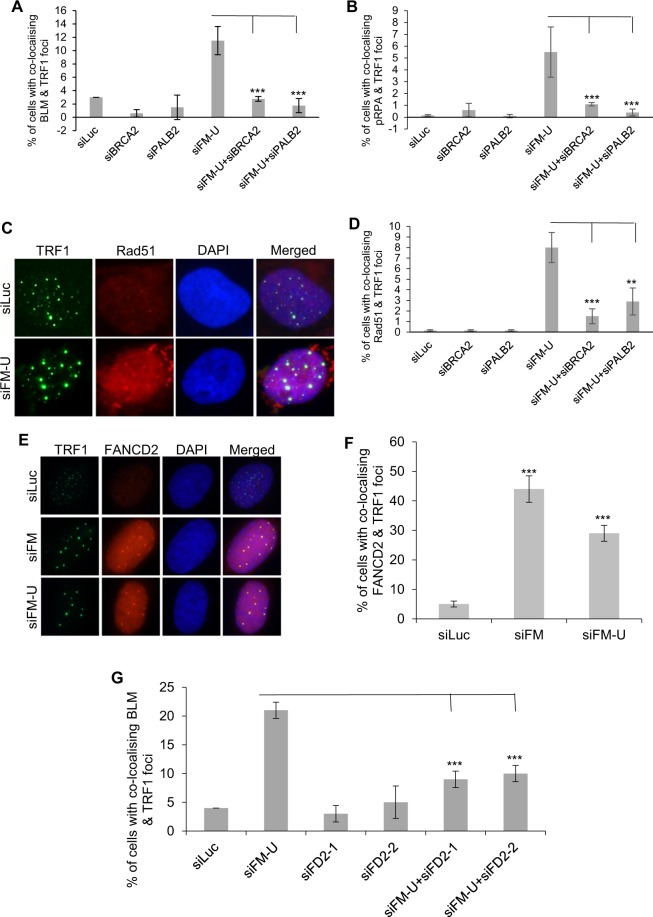
(**A–D**) BRCA2 and PALB2 facilitate ssDNA formation and homologous recombination at telomeres in FANCM deficient ALT cells. siRNA transfected U2-OS cells were co-stained with antibodies recognizing BLM and TRF1, or pRPA and TRF1, or Rad51 and TRF1. (**E–G**) FANCD2 is recruited to the telomeres in FANCM deficient cells and facilitates the recruitment of BLM. siRNA transfected U2-OS cells were co-stained with antibodies recognizing FANCD2 and TRF1, or BLM and TRF1. More than three co-localized FANCD2 and TRF1 foci were counted. All error bars are standard deviation of the mean obtained from three different experiments. Standard two-tailed Student’s t-test: *p < 0.05, **p < 0.01, ***p < 0.001, ns – not significant.

Since multiple HR proteins affect the CC formation (Table 1), including BRCA2, PALB2 and Rad51, we further investigated their functions in FANCM deficient ALT. As shown in Fig. 3A–D, depletion of BRCA2 or PALB2 attenuated the telomeric BLM, pRPA and Rad51 foci formation in FANCM deficient ALT, suggesting that both BRCA2 and PALB2 are involved in multiple steps of the replication stress response at ALT telomeres, including ssDNA formation and Rad51-dependent strand invasion.

A previous study suggests that FANCD2 counters the function of BLM during telomere replication and recombination in ALT cells^41^. In our targeted screening, we found that FANCD2 is one of the DDR genes, depletion of which most severely affected the CC formation in FANCM deficient U-2 OS (Table 1). Consistent with the previous report^41^, we also found that depletion of FANCD2 mildly stimulates the CC formation in FANCM proficient cells. To further investigate the role of FANCD2 in the ALT pathway, we performed immunostaining to determine whether FANCD2 can be recruited to the replication stressed telomeres. Interestingly, in FANCM deficient cells, we observed a pronounced formation of FANCD2 foci, which co-localize with the large and intense TRF1 foci, suggesting that FANCD2 is actively recruited to the replication stressed telomeres (Fig. 3E,F). Depletion of FANCD2 using two different siRNA attenuated the formation of telomeric BLM foci in FANCM deficient ALT cells, but not the pChk1, or pRPA foci (Figs. 3G and S4). These data suggest that FANCD2 may partially contribute to the recruitment of BLM to the replication stressed ALT telomeres, but is dispensable for the checkpoint activation and ssDNA formation at the damaged telomeres.

### In FANCM deficient ALT cells, accumulation of TERRA R-loops at telomeres leads to heightened replication stress response and upregulation of C-circles formation

We recently reported that depletion of FANCM in multiple ALT cell lines leads to robust replication stress at their telomeres^39^. We proposed that the potential causes for the replication stress at FANCM deficient ALT telomeres may include accumulation of unresolved HR intermediates, G4, or TERRA R-loops^42^. Arora and colleagues showed that TERRA R-loops contribute to the recombinogenic nature of ALT telomeres and regulate their maintenance^43^. Intriguingly, Schwab and colleagues demonstrated that recombinant FANCM is capable of unwinding generic R-loops *in vitro*^30^. We thus hypothesized that, in FANCM deficient ALT cells, TERRA R-loops accumulate at telomeres and block the progression of replisomes, leading to the replication stress response.

**Figure 4:**
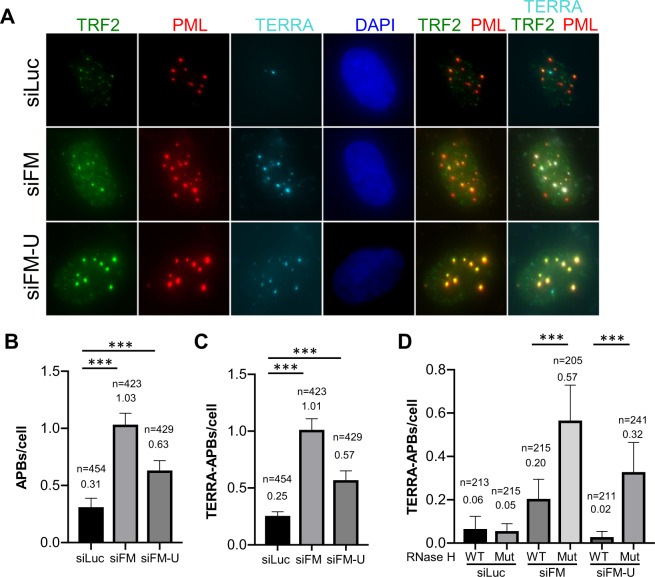
Depletion of FANCM leads to TERRA R-loop accumulation at the ALT telomeres. (**A**) siRNA transfected U2-OS cells were co-stained with TERRA probe and antibodies recognizing PML and TRF2. (**B**,**C**) The number of APBs and TERRA-associated APBs were identified and counted by the colocalization of PML with TRF2, or both TRF2 and TERRA. (**D**) U-2 OS cells overexpressing either wild-type (WT) RNase H1 or mutant (Mut) RNase H1were transfected with siRNA and then co-stained with TERRA probe and antibodies recognizing PML and TRF2. Values in B to D are the mean with 95% of confidence interval. Data was collected from two biological replicates. Standard two-tailed Student’s t-test: ***p < 0.001. n = number of cells.

To test this hypothesis, we first performed immuno-RNA Fluorescent *In Situ* Hybridization (FISH) to detect the TERRA associated APBs. As shown in Figs. 4A–C and S5A,B, we observed a significant increase of TERRA associated APBs in FANCM depleted cells. When the wild-type RNase H1, a ribonuclease that cleaves the RNA molecule within a DNA-RNA hybrid, but not the mutant RNase H1, was overexpressed in these cells, TERRA associated ABPs were attenuated (Figs. 4D and S5C,D).

**Figure 5:**
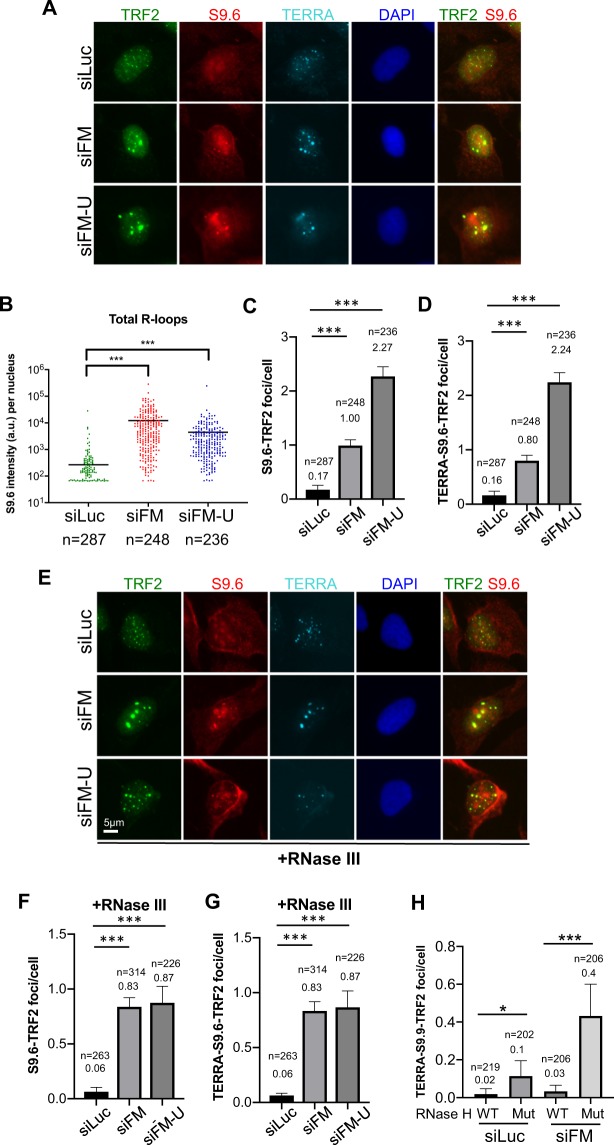
S9.6 positive R-loops accumulate at telomeres in FANCM deficient ALT cells. U-2 OS cells were first transfected with siRNA. (**A–D**) Immuno-RNA FISH staining for TERRA, S9.6 and TRF2 were performed and quantified. The total intensity of S9.6 staining per nucleus was quantified in (**B**). The number of S9.6 foci that co-localize with the TRF2 foci was quantified in (**C**). The number of S9.6 foci that co-localize with the TRF2 and TERRA was quantified in (**D**). (**E**–**G**) RNase III was added to remove double-stranded RNA signals prior to immune-RNA FISH for TERRA, S9.6 and TRF2. The number of S9.6 foci that co-localize with the TRF2 foci was quantified in (**F**). The number of S9.6 foci that are co-localize with the TRF2 and TERRA was quantified in (**G**). (**H**) U-2 OS cells overexpressing either wild-type (WT) RNase H1 or mutant (Mut) RNase H1 were transfected with siRNA and then co-stained with TERRA probe and antibodies recognizing S9.6 and TRF2. The number of S9.6 foci that are colocalized with the TRF2 and TERRA was quantified. Values in B to D and F to H are the mean with 95% of confidence interval. Data were collected from two biological replicates (**B–D,F,G**). Standard two-tailed Student’s t-test: *p < 0.05. ***p < 0.001. n = number of cells.

To further verify the R-loop accumulation at telomeres in FANCM depleted cells, we performed an immunofluorescent staining using the monoclonal antibody S9.6 to detect the DNA:RNA hybrid^31^. At the same time, cells were also co-stained with an antibody recognizing TRF2 and the probe for TERRA RNA (Fig. 5A). We found that not only the total R-loops were elevated in FANCM deficient cells as reported previously (Fig. 5B)^30^, but the S9.6-TRF2 double positive R-loops and the TERRA-S9.6-TRF2 triple positive R-loops were also significantly increased by 5–10 fold (Figs. 5C,D and S6A,B). Notably, most of the S9.6-TRF2 co-localized foci were also stained positive with TERRA. The results did not change when cells were pre-treated with RNase III prior to S9.6 and TERRA staining to remove the double-stranded RNA (dsRNA) signals (Figs. 5E–G and S6C,D). When cells were pre-treated with RNase H prior to immuno-RNA-FISH, the intensity of S9.6 staining was greatly reduced, suggesting that S9.6 antibody specifically stains DNA-RNA hybrids as previously reported (Fig. S6E)^31^. Furthermore, overexpressing of RNase H1 attenuated the checkpoint activation, CC formation and TERRA R-loop accumulation in FANCM depleted cells (Figs. 5H and S6F–H).

Collectively, the data presented here strongly support our hypothesis that in ALT cells, FANCM disrupts the TERRA R-loop accumulation at telomeres to avoid the stalling/collapsing of replication forks during telomere replication.

### The helicase/translocase activity of FANCM and, to a lesser degree, its interaction with FAAP24 are important for its function at ALT telomeres

**Figure 6:**
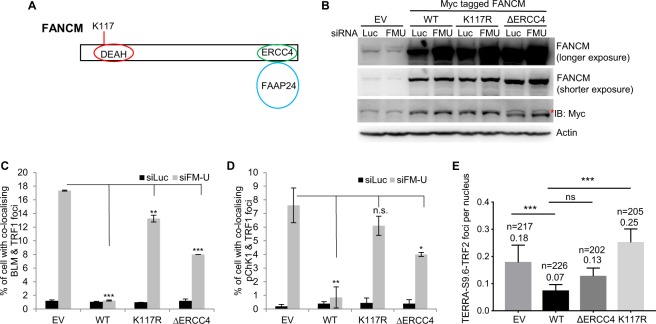
The helicase domain and the FAAP24 interacting domain of FANCM facilitate the disruption of R-loop formation at ALT telomeres. (**A**) A diagram of FANCM domain structure. (**B**) The expression level of FANCM in empty vector (EV) transfected U-2 OS cells, or U2-OS cells stably expressing wild-type FANCM (WT), or FANCM mutants (K117R and ΔERCC4). The red asterisk indicates the cross-reaction band from the anti-Myc antibody. (**C**,**D**) siRNA transfected U2-OS cells were co-stained with antibodies recognizing BLM and TRF1, or pChk1 and TRF1. All error bars are standard deviation of the mean obtained from two different experiments. Standard two-tailed Student’s t-test: *p < 0.05, **p < 0.01, ***p < 0.001, ns – not significant. (**E**) The number of TERRA R-loops per nucleus was counted in various cell lines. Values are the mean with 95% of confidence interval. Standard two-tailed Student’s t-test: ***p < 0.001. n = number of cells.

As mentioned above, human FANCM contains a conserved DEAH box at its N-terminus and belongs to the SF2 family of helicases/translocases (Fig. 6A)^26^. At its C-terminus, FANCM contains a degenerated ERCC4 nuclease domain, which is required for its interaction with FAAP24 (Fig. 6A)^34^.

To investigate which domain of FANCM is important for its function at the ALT telomeres, we generated U2-OS cell lines with an integrated empty vector (EV), or stably overexpressing the wild-type FANCM (WT), or the helicase/translocase mutant (K117R), or the ERCC4 deletion mutant (ΔERCC4)^44^. siFM-U, which targets the 3′ UTR of endogenous FANCM mRNA, was used to deplete the endogenous FANCM, but not the exogenously overexpressed FANCM-WT, nor FANCM-K117R, nor FANCM- ΔERCC4 (Fig. 6B). Remarkably, overexpression of FANCM-WT completely suppressed the formation of BLM and pChk1 foci in siFM-U transfected cells, while the overexpression of FANCM-ΔERCC4 had a partial effect (Fig. 6C,D). In contrast, overexpression of FANCM-K117R only mildly suppressed the foci formation of BLM, but failed to suppress the pChk1 foci formation (Fig. 6C,D). Consistent with these observations, overexpression of FANCM-WT, but not FANCM-ΔERCC4 and FANCM-K117R, suppressed the basal TERRA R-loop formation (Fig. 6E).

Taken together, our data indicate that the translocase/helicase activity of FANCM is required for the removal of TERRA R-loops and the inhibition of replication stress at ALT telomeres. Its interaction with FAAP24 is likely important for those functions as well.

## Discussion

Though the ALT pathway was discovered over twenty years ago^12^, the detailed mechanism of how ALT is initiated and maintained remains poorly defined^15,45^. It was shown previously that, in comparison to TEL+ cells, ALT cells are more prone to experiencing spontaneous DNA damage at their telomeres^46^. However, the exact cause(s) of the spontaneous DNA damage and how ALT cells cope with these damages warrant further investigation.

We recently reported that depletion of FANCM in multiple ALT cell lines induces pronounced replication stress, which primarily takes place at their telomeres^39^. We thus named this system M-SAT (FANCM deficiency-induced replication stress at ALT telomeres). In the same study, we demonstrated that alleviating the replication stress in the M-SAT system requires coordinated actions of multiple DDR proteins, including BLM, BRCA1, ATR, Chk1, and Rad51. Built on these previous findings, we showed here that depletion of FANCM instigated a dramatic increase of APBs and CC in ALT cells (Figs. 1, 4A–C and S1). Similar observations have been independently reported recently by two other groups^44,47^. Together, these data suggest that FANCM plays a critical role in suppressing various ALT properties. Among the different ALT properties, CC formation is considered to be the most specific and quantifiable biomarker^16^. However, the molecular mechanism of how CC is generated and regulated is still largely unknown. To take advantage of this robust CC induction system (the M-SAT system), we performed a targeted screening and identified 12 different DDR proteins that promote the CC formation, including checkpoint kinases, the dissolvase complex, and proteins involved in HR and BIR. Our screening results thus suggest that CC is likely produced at a later stage of the replication stress response at ALT telomeres. Finally, we demonstrated that depletion of FANCM in ALT cells leads to a pronounced accumulation of TERRA R-loops at telomeres. Taken together, we propose that, at ALT telomeres, FANCM-FAAP24 actively disrupt the TERRA R-loops, prevent the stalling/collapsing of replication forks during telomere replication and attenuate the formation of CC (Fig. S7).

### FANCM functions as a helicase/translocase to disrupt the TERRA R-loops and prevent the pausing/stalling of replisomes at ALT telomeres

Though human FANCM contains a classic SF2 helicase domain at its N-terminus, it acts primarily on branched DNA structures that mimic stalled replication forks or Holiday junction intermediates, and promotes fork branch migration^29^. Schwab and colleagues showed that recombinant human FANCM is also able to resolve generic R-loops via its branch migration activity *in vitro*^30^. Using the S9.6 monoclonal antibody in the immunofluorescent staining, they were able to detect mild increase of R-loop formation in FANCM deficient cells. However, where the R-loops are formed in the genome is unknown. Here, using the TERRA specific probe, S9.6, and telomeric markers, we detected a 5- to 10-fold increase of TERRA R-loops at telomeres in FANCM deficient ALT cells. Additionally, when wild-type RNase H1 was overexpressed, the accumulation of TERRA R-loops, the activation of DNA damage checkpoint and the formation of CC were significantly attenuated. Finally, we showed that the helicase/translocase activity of FANCM and its interaction with FAAP24 are important for its functions at telomeres. Our data thus indicate that, in FANCM proficient ALT cells, FANCM-FAAP24 actively promotes the resolution of TERRA R-loops at telomeres to prevent replication forks from pausing/stalling. Though we are able to detect both FANCM and FAAP24 at the ALT telomeres^39^, currently, we cannot distinguish whether they are part of the replisome at telomeres, or are actively recruited there whenever and wherever the replication fork pauses or stalls. A recent study in budding yeast showed that the budding yeast homolog of FANCM, Mph1, can be recruited to the accumulated R-loops induced by either the deletion of RNase H, or THO-complex mutants, or shorter telomeres, suggesting that the function of FANCM in resolving R-loops at telomeres is highly conserved^48^.

### Rad51-dependent BIR likely plays an important role to repair/re-start the stalled/collapsed replication forks at ALT telomeres

BIR is a specialized homology-dependent DNA synthesis pathway. BIR is activated when a one-ended DSB persists and invades a double stranded DNA homologous to the DNA sequences at the break. BIR is mostly studied in the budding yeast, *S. cerevisiae*^49,50^. Well-established BIR factors in yeast include Pif1 (a DNA helicase), Pol δ (a DNA polymerase), and Pol32 (one of the accessory factors of Pol δ). Pol32 is not required for normal S phase DNA synthesis, but it is essential for DNA synthesis via BIR during DNA repair. Therefore, the dependence on Pol32 signifies the activation of BIR. It has also been well established that, in *S. cerevisiae*, there exist a Rad51-dependent BIR and a Rad51-independent BIR, and both require Rad52. In the absence of *S. cerevisiae* telomerase, BIR becomes essential for both Type I and Type II Survivors^24,51^. Type I survivors maintain their DNA ends by recombining and amplifying Y’ subtelomeric sequences and rely on the Rad51-dependent BIR. Type II survivors, on the other hand, adopt the Rad51-independent BIR and can acquire longer telomeres.

In recent years, studies from three different groups also implicated BIR in the ALT pathway in humans. In a study by Roumelioti and colleagues, they showed that conservative DNA synthesis exists at ALT telomeres^20^. Most importantly, they showed that depletion of PolD3, the human homolog of *S. cerevisiae* Pol32, compromised the conservative telomeric DNA replication and produced shorter telomeres. In another study by Dilley and colleagues, they showed that both PolD3 and Pol δ, but not Polη, Polζ, and Rad51, are required for the DSB-induced telomere synthesis^18^. In another study by Min and colleagues, they found that heightened telomeric replication stress in ALT cells induces mitotic DNA synthesis (MiDAS) at telomeres, which is also mediated by BIR and is dependent on Rad52, but not Rad51^19^.

In our previous study, we showed that BLM and BRCA1 actively recruit Rad51 to the replication stressed ALT telomeres^39^. Here we reported that BRCA2 and PALB2 are also involved in recruiting Rad51 to the replication stressed ALT telomeres. In addition, we showed that depletion of Rad51 attenuated the CC formation in FANCM deficient ALT cells. Similar to a recent report by Zhang and colleagues, we also found that Rad52 is dispensable for the CC formation in FANCM deficient ALT^52^. In mammals, BRCA2 has been proposed to play an overlapping role with Rad52^53^. Indeed, depletion of BRCA2 in FANCM deficient ALT also affects CC formation, suggesting that in the M-SAT system, BRCA2 likely substitutes Rad52 to facilitate the strand invasion by Rad51. In our targeted screening, we also identified BIR as a major pathway that affects the CC formation. We showed that depletion of the two important BIR factors, PolD3 and Pol δ, severely attenuated CC formation in FANCM deficient ALT cells, which is consistent with the finding in yeast that overexpression of MPH1, the yeast homolog of FANCM, suppresses BIR^25^. Though we did not observe any pronounced effect on CC formation when PolD3 is depleted using siRNA in FANCM proficient U-2 OS cells, Dilley and colleagues did observe a mild effect when PolD3 is permanently deleted using CRISPR, suggesting that PolD3 may still play a role in the long-term maintenance of CC formation in ALT cells^18^. In addition to Pol δ, we found that a translesion DNA polymerases, Polη, is also important for the dramatic increase of CC in FANCM deficient ALT cells. Previously, Garcia-Exposito and colleagues identified Polη as an ALT telomere binding protein in a BioID proteomic screening and proposed that it may be involved in initiating DNA synthesis at ALT telomeres^54^. In light of our findings, we propose that, in FANCM deficient ALT, BIR is activated to repair and re-start the stalled/collapsed replication forks at their telomeres due to the accumulation of TERRA R-loops. In the first step of BIR, BRCA2 and PALB2, instead of Rad52, facilitate the strand invasion by Rad51. Subsequently, DNA synthesis is initiated by Polη and further extended by Pol δ (Fig. S7)

### In the absence of telomerase, heightened replication stress at telomeres may facilitate the initiation of ALT pathway during cellular transformation

Compared to TEL + cells, ALT cells share certain distinctive features, including increased frequency of tSCE, increased formation of APBs and TIFs, and the appearance of a large amount of ECTR, such as CC and GC^14^. In addition, ALT telomeres tend to be more heterogeneous and some of them can be quite long^12^. Among these ALT features, CC formation is established as the most robust, specific, and quantifiable biomarker^16^. However, the etiology and functional significance of CC is still unclear. Here, we showed that transient depletion of FANCM leads to TERRA R-loop accumulation, replication stress response, and dramatic upregulation of CC formation. Conversely, overexpression of RNase H1 attenuates TERRA R-loop formation, replication stress response, and the CC formation. Depletion of multiple BIR proteins also attenuates CC formation, suggesting that the upregulation of CC in the M-SAT system is likely a downstream event of BIR. Replication stress has been proposed to be the root cause of various ALT properties^14^. For example, when replication forks are stalled or collapsed at ALT telomeres, they are then clustered together and form the APBs to facilitate the repair processes. The one-ended or two-ended DNA breaks derived from the stalled/collapsed replication forks manifest as TIFs and promote tSCE. Cleavage or processing of the intermediates formed during the repair and re-start of the stalled/collapsed replication forks via BIR produce CC and other ECTRs, most likely as the by-products. DNA synthesized via BIR can be quite long^18^, thus produces the heterogeneous and super long telomeres.

Among the eighteen DDR proteins tested, the deficiency of eleven proteins affects CC formation only in the FANCM deficient ALT cells, while the deficiency of only one protein, BLM, affects CC formation in both FANCM deficient ALT cells as well as FANCM proficient ALT cells. Surprisingly, we did not find any protein that affects the CC formation only in FANCM proficient ALT cells. Collectively, these results suggest that the pathways required to initially establish the ALT (e.g., as modeled by the FANCM deficient ALT cells) may be different with the pathways that are required to maintain the ALT (e.g., as modeled by the FANCM proficient ALT cells). Alternatively, the initiation and the maintenance of ALT may use the same pathways, but because the replication stress is much lower in the well-established, or the maintenance stage, ALT cells, the CC assay is not sensitive enough to detect the changes.

Based on the results presented here and from other studies^14,15,23,44,47^, it is tempting to speculate the general processes by which ALT cancers are derived from telomerase negative cells: loosened chromatin environment at telomeres leads to increased transcription of TERRA, thus increased chances of replication fork pausing/stalling due to the frequently unresolved TERRA R-loops. Occasionally, a collapsed replication fork produces a one-ended DSB and subsequently activates BIR, which repairs and re-starts the collapsed replication forks, thus the full activation of the ALT pathway, to escape telomere shortening induced cell cycle arrest and cell death.

### Targeting the replication stress response for ALT cancer therapy

Previously, ATR was shown to be critical for the survival of ALT cancers^55^. In our recent studies^39^, we found that co-depletion of FANCM and BLM, or FANCM and BRCA1, induces synthetic lethality in ALT cells but not TEL+ cells. Though the exact mechanism is still unclear, we speculate that, in the absence of BLM or BRCA1, the stalled/collapsed replication forks at telomeres in FANCM deficient ALT cells failed to be repaired, which then eventually leads to cell death. Here, we have uncovered additional factors that are required for the ALT cells to cope with the replication stress response at their telomeres, and many of them are enzymes, including TOP3A, Pol δ and Polη. Inhibitors targeting these enzymes could potentially be more efficacious in treating ALT cancers.

## Methods

### Cell lines and cell culture

U2-OS, Saos-2, HeLa and MG63 cells were purchased from ATCC. All cells were grown in D-MEM supplemented with 10% fetal bovine serum (FBS) and penicillin and streptomycin and cultivated at 37 °C in a humidified incubator with 5% CO_2_. U2-OS cell lines with overexpression of wild-type RNase H1 and mutant RNase H1 (enzyme dead) were generated by transfection of pICE-RNaseH1-WT-NLS-mCherry (Addgene #60365) and pICE-RNaseH1-D10R-E48R-NLS-mCherry (Addgene#60367) and selected with puromycin.

### Chemicals and plasmids

The empty vector, pcDNA3.1, was purchased from Invitrogen. The RNAse H1 overexpressing plasmids, pcDNA3.1-RNase H1, was generously provided by Dr. Advaitha Madireddy (Robert Wood Johnson Medical School).

### Telomere restriction fragment (TRF) assay

Cells were transfected twice with different siRNA. 24 hours later, genomic DNA from 3–5 × 10^5^ cells was extracted using QIAamp DNA Blood Mini Kit (Qiagen, 51106). 2 μg genomic DNA were digested with Hinf I (NEB, R0155) and Rsa I (NEB, R0167) at 37 °C overnight and then run on a 0.8% agarose gel in 1X TAE. After DNA was transferred to an Amersham Hybond-N + membrane, the membrane was then processed, probed, and developed using the TeloTAGGG Telomere Length Assay kit (Roche, 12-209-136-001). Images were taken with an Amersham Imager 600.

### C-circle assay

Cells were transfected twice with different siRNA. 24 hours later, genomic DNA from 3–5 × 10^5^ cells was extracted using QIAamp DNA Blood Mini Kit (Qiagen, 51106). 4 μg genomic DNA were digested with Alu I (NEB, R0137L) and Mbo I (NEB, R0147L) at 37 °C for 2 hours and then purified using Qiagen PCR Purification Kit (Qiagen, 28106). 40 ng of Alu I and Mbo I digested DNA were used for the Phi29 DNA polymerase reaction (30 °C for 8 hours and then 65 °C for 20 min). All of the PCR reaction mixtures were loaded onto the Amersham Hybond-N + membrane using the Bio-Rad Bio-Dot and Bio-Dot SF Microfiltration apparatus. The membrane was cross-linked using the UV Stratalinker at energy 1200. The membrane was then processed, probed, and developed using the TeloTAGGG Telomere Length Assay kit (Roche, 12-209-136-001). Images were taken with an Amersham Imager 600 and quantified using the NIH ImageJ.

### Antibodies used for immunoblotting

Actin (Santa Cruz, sc-1616); BLM (Bethyl, A300-110A); RMI1 (Bethyl, A300-631A); TOP3A (ProteinTech, 14525-1); ATR (Santa Cruz, sc-1887); Chk1 (Santa Cruz, sc-8408); PolD3 (Abnova, H00010714-M01); PolD1 (Bethyl, A304-005A); PolH (Bethyl, A301-231A); BRCA1 (EMD/Calbiochem, OP92); BRCA2 (EMD/Calbiochem, OP95); PALB2 (Bethyl, IHC-00251); Rad51 (Santa Cruz, sc-8349); Rad52 (Santa Cruz, sc-365341); Mre11 (Novus, NB100-142); Smc5 (Bethyl, A300-236A); Smc6 (Bethyl, A300-237A); FANCD2 (Novus, 100-182); RNas H1 (ProteinTech, 15606-1); mCherry (GeneTex,GTX128508), GAPDH (Cell Signaling, #2118). Myc (Santa Cruz, sc-40). Antibodies recognizing FANCM, FAAP24, MHF1, and MHF2 are generously provided by Dr. Xiaodong Wang. The antibody recognizing CtIP was generously provided by Richard Baer.

### Immunofluorescent staining

U2-OS cells were transfected twice with different siRNA and then re-plated on coverslips. Cells were then used for immunostaining 72 hours later. Briefly, cells were first fixed with 3% paraformaldehyde containing 2% sucrose for 10 min, then were permeablized with Triton X-100 solution on ice for 5 min, and stained with different primary antibodies (as indicated later) and the appropriate Alexa-488 (Invitrogen) and Alexa-546 (Invitrogen) conjugated secondary antibodies. Images were taken with an Olympus upright fluorescent microscope. Antibodies used for immunofluorescent staining include: PML (Bethyl, A310-167A); Chk1-pS345 (Cell Signaling, 2348); RPA32-pS4pS8 (Bethyl, A300-245A); BLM (Bethyl, A300-110A); BRCA1 (Bethyl, A301-377A); Rad51 (Santa Cruz, sc-8349); TRF1 (abcam, ab10579); TRF2 (Millipore, 05-521 and Novus, NB110-57130); FANCD2 (Novus, 100-182); S9.6 (Millipore, MABE1095).

### Immuno-RNA-FISH

U-2 OS cells grown on cover slips were washed with cold PBS and treated with CSKT (10 mM PIPES, pH 6.8, 100 mM NaCl, 3 mM MgCl_2_, 0.3 M sucrose, 0.5% Triton X-100, adjust to pH 6.8) for 10 minutes on ice. Cells were fixed in 4% paraformaldehyde at RT and stored at 70% ethanol at −20 °C. After washing with cold PBS, cells were incubated with blocking solution (1% BSA/PBS with 1 mM EDTA and 0.8 U/μl of RNase inhibitor) at 4 °C for 1 hr. Cells were then incubated with primary antibodies in blocking solution at 4 °C overnight, and washed with 0.2% Tween20/PBS three times at 4 °C. Antibodies were used against TRF2 (Novus, NB110-57130) and PML (Santa Cruz, sc-966). Followed by incubation of secondary antibodies (in blocking solution) at 4 °C for 2 hours, cells were washed with PBS three times and fixed in 2% paraformaldehyde for 10 minutes. RNA FISH was then performed after immunostaining. TERRA oligo probes ((TAACCC)_7_-Alexa-647-3′) for RNA-FISH were mixed at the final concentration of 0.5 pmol/μl in hybridization solution (50% formamide, 2 × SSC, 2 mg/ml BSA, 10% Dextran Sulfate-500 K). Hybridization was carried out at 42 °C overnight for RNA FISH. Cells were washed with 2 × SSC/50% formamide for 5 min three times at 44 °C, and then washed with 2 × SSC for 5 min twice at 44 °C. Images were captured using Olympus IX83 inverted microscopy with various Z-sections and then were compiled to 3D images to calculate APB and TERRA-associated APB foci. ABP foci were determined by the extensive TRF2 staining (>3 fold increase compared to the average signal) with PML staining.

## Supplementary information

Supplemental information
