# Supplementary material for: FANCM suppresses DNA replication stress at ALT telomeres by disrupting TERRA R-loops
Source: Sci Rep. 2019 Dec 13;9:19110. doi: 10.1038/s41598-019-55537-5 (PMC6911001; doi:10.1038/s41598-019-55537-5)
Supplement: Supplementary file 1 — Supplemental information [file 41598_2019_55537_MOESM1_ESM.pdf]

# FANCM suppresses DNA replication stress at ALT telomeres by disrupting TERRA R-loops

Xiaolei Pan,[1] Yun Chen,[2] Beena Biju,[1] Naveed Ahmed,[1] Joyce Kong,[1], Marti Goldenberg,[1] Judy Huang,[1] Nandakumar Mohan,[1] Stephanie Klosek,[1] Kian Parsa,[1] Chia-Yu Guh,[2] Robert Lu,[3] Hilda A Pickett,[3] Hsueh-Ping Chu,[2,*] and Dong Zhang[1,*]

**SUPPLEMENTAL TABLE AND FIGURE LEGENDS**:

**Supplemental Table S1:** siRNA used in this study

**Fig. S1**: Depletion of FANCM in Saos-2, but not HeLa and MG63 cells, induces pronounced C-circles formation. Saos-2, or HeLa, or MG63 cells were transfected with siRNA targeting either luciferase (siLuc) or FANCM (siFM and siFM-U). Genomic DNA was extracted and used for C-circle assay. "–Φ" indicates samples with no Phi(Φ)29 DNA polymerase added. The amount of C-circles in siLuc transfected cells is used to normalize C-circles in other siRNA transfected cells. All error bars are standard deviation of the mean obtained from two different experiments. Standard two-tailed Student's t-test: **$p$<0.01.

**Fig. S2**: Depletion of the FANCM complex has no pronounced effects on the telomere length. U-2 OS cells were transfected with siRNA targeting either luciferase (siLuc), FAAP24 (siFAPP24-1 and siFAAP24-2), FANCM (siFM and siFM-U), MHF1, MHF2, or both MHF1 and MHF2. Genomic DNA was extracted from the siRNA transfected cells and digested with Hindf I and Rsa I and then run on a 0.8% agarose gel.

**Fig. S3**: Knockdown efficiency of different siRNA in U2-OS cells.

**Fig. S4**: Depletion of FANCD2 does not affect checkpoint activation and ssDNA formation at telomeres in FANCM deficient cells. siRNA transfected U2-OS cells were co-stained with antibodies recognizing pChk1 and TRF1, or pRPA and TRF1. All error bars are standard deviation of the mean obtained from three different experiments. Standard two-tailed Student's t-test: n.s., not significant.

**Fig. S5**: Histogram graphs show the percentage of cells with the indicated number of ABP foci per nucleus (A) and the percentage of cells with the indicated number of TERRA associated APB foci (B, D). siRNA transfected cells were stained with TERRA probe, and antibodies recognizing PML or TRF2. (C) U2-OS cells stably express either wild-type (WT) RNase H1 or

mutant (Mut) RNase H1. The *p* values were calculated by two-tailed Student's t-test. n= number of cells.

**Fig. S6**: Histogram graphs show the percentage of cells with the indicated number of telomeric R-loops foci (S9.6 and TRF2 colocalization) per nucleus (A, C) and the percentage of cells with the indicated number of TERRA R-loops (TERRA, S9.6 and TRF2 colocalization) foci per nucleus (B, D). RNase III was added to siRNA transfected cells to remove double-stranded RNA signals prior to immune-RNA FISH for TERRA, S9.6 and TRF2 (C, D). The *p* values in A to D were calculated by two-tailed Student's t-test. n= number of cells. Pretreatment of RNase H prior to S9.6 staining reduces the overall S9.6 signals in FANCM knockdown (siFM) cells (E), indicating the specificity of the S9.6 signals for DNA-RNA hybrids. (F to G) siRNA transfected U2-OS cells were co-transfected with either empty vector or a plasmid expressing RNase H1. The expression level of RNase H1 is shown in (F). One set of cells were co-stained with antibodies recognizing pChk1 and TRF1 (G). Another set of cells were used for C-circle analysis (H). All error bars in F and G are standard deviation of the mean obtained from two different experiments. Standard two-tailed Student's t-test: *$p<0.05$, **$p<0.01$.

**Fig. S7**: A model of TERRA R-loops induced replication stress response at ALT telomeres. In FANCM deficient ALT cells, TERRA R-loops accumulate at telomeres, which then lead to pausing/stalling of replication forks. Repair/re-start of the stalled replication forks via BIR leads to the up-regulation of C-circles formation.

**Supplemental Table S1:** siRNA used in this study

| | Name | Sequence | References |
|---|---|---|---|
| Control | Luc | UCGAAGUAUUCCGCGUACGUU | (Pan et al., 2017) |
| FANCM complex | FANCM | AAGCUCAUAAAGCUCUCGGAA | (Pan et al., 2017) |
| | FANCM-U | AAAGACCUCUCACAAUAUU | (Pan et al., 2017) |
| | FAAP24-1 | CCGGAUGAGUGAACAAUACUU | (Pan et al., 2017) |
| | FAAP24-2 | AUUUUCGAGGAUGGCUUGACA | (Pan et al., 2017) |
| | MHF1 | DHARMACON, L-032895-01 | (Pan et al., 2017) |
| | MHF2 | DHARMACON, L-016829-01 | (Pan et al., 2017) |
| Dissolvase complex | BLM | CCCACUACUUUGCAAGUAA | (Pan et al., 2017) |
| | TOP3A | ACAUCGGGUUUGAGAUUAU | (Yang et al., 2012) |
| | RMI1 | AGCCUUCACGAAUGUUGAU | (Yang et al., 2012) |
| Checkpoint kinases | ATR | CCUCCGUGAUGUUGCUUGA | (Pan et al., 2017) |
| | Chk1 | AAGCGUGCCGUAGACUGUCCA | (Pan et al., 2017) |
| BIR | Pol D3 | TGGCATTATGTCTAGGACTAA | (Costantino et al., 2014) |
| DNA polymerase | Pol D1 | GAGAGAGCAUGUUUGGGUA | (Dilley et al., 2016) |
| | Pol H | CTGGTTGTGAGCATTCGTGTA | (Dilley et al., 2016) |
| HR | BRCA1 | GAAGGAGCUUUCAUCAUUC | (Pan et al., 2017) |
| | BRCA2 | GGGAAACACUCAGAUUAAAUU | (Ahrabi et al., 2016) |
| | PALB2 | DHARMACON, D-012928-04 | (Orthwein et al., 2015) |
| | Rad51 | UGUAGCAUAUGCUCGAGCG | (Dilley et al., 2016) |

| | | | |
|---|---|---|---|
| | Rad52 | Qiagen, SI03035123 | (Sotiriou et al., 2016) |
| DNA end resection nuclease | CtIP | GCUAAAACAGGAACGAAUC | (Pan et al., 2017) |
| | Mre11 | GGAGGUACGUCGUUUCAGA | (Pan et al., 2017) |
| Cohesion-like complex | Smc5 | GAAGCAAGAUGUUAUAGAA | (Min et al., 2017) |
| | Smc6 | AGAGCGGCUUACUGAACUA | (Min et al., 2017) |
| Other FA | FANCD2-1 | GGAGAUUGAUGGUCUACUA | (Kais et al., 2016) |
| | FANCD2-2 | CAGAGUUUGCUUCACUCUCUA | (Murina et al., 2014) |

**References**


Ahrabi, S., Sarkar, S., Pfister, S.X., Pirovano, G., Higgins, G.S., Porter, A.C., and Humphrey, T.C. (2016). A role for human homologous recombination factors in suppressing microhomology-mediated end joining. Nucleic Acids Res *44*, 5743-5757.

Costantino, L., Sotiriou, S.K., Rantala, J.K., Magin, S., Mladenov, E., Helleday, T., Haber, J.E., Iliakis, G., Kallioniemi, O.P., and Halazonetis, T.D. (2014). Break-induced replication repair of damaged forks induces genomic duplications in human cells. Science *343*, 88-91.

Dilley, R.L., Verma, P., Cho, N.W., Winters, H.D., Wondisford, A.R., and Greenberg, R.A. (2016). Break-induced telomere synthesis underlies alternative telomere maintenance. Nature *539*, 54-58.

Kais, Z., Rondinelli, B., Holmes, A., O'Leary, C., Kozono, D., D'Andrea, A.D., and Ceccaldi, R. (2016). FANCD2 Maintains Fork Stability in BRCA1/2-Deficient Tumors and Promotes Alternative End-Joining DNA Repair. Cell Rep *15*, 2488-2499.

Min, J., Wright, W.E., and Shay, J.W. (2017). Alternative Lengthening of Telomeres Mediated by Mitotic DNA Synthesis Engages Break-Induced Replication Processes. Mol Cell Biol *37*.

Murina, O., von Aesch, C., Karakus, U., Ferretti, L.P., Bolck, H.A., Hanggi, K., and Sartori, A.A. (2014). FANCD2 and CtIP cooperate to repair DNA interstrand crosslinks. Cell Rep *7*, 1030-1038.

Orthwein, A., Noordermeer, S.M., Wilson, M.D., Landry, S., Enchev, R.I., Sherker, A., Munro, M., Pinder, J., Salsman, J., Dellaire, G., *et al.* (2015). A mechanism for the suppression of homologous recombination in G1 cells. Nature *528*, 422-426.

Pan, X., Drosopoulos, W.C., Sethi, L., Madireddy, A., Schildkraut, C.L., and Zhang, D. (2017). FANCM, BRCA1, and BLM cooperatively resolve the replication stress at the ALT telomeres. Proc Natl Acad Sci U S A *114*, E5940-E5949.

Sotiriou, S.K., Kamileri, I., Lugli, N., Evangelou, K., Da-Re, C., Huber, F., Padayachy, L., Tardy, S., Nicati, N.L., Barriot, S., *et al.* (2016). Mammalian RAD52 Functions in Break-Induced Replication Repair of Collapsed DNA Replication Forks. Mol Cell *64*, 1127-1134.

Yang, J., O'Donnell, L., Durocher, D., and Brown, G.W. (2012). RMI1 promotes DNA replication fork progression and recovery from replication fork stress. Mol Cell Biol *32*, 3054-3064.

# Fig S1

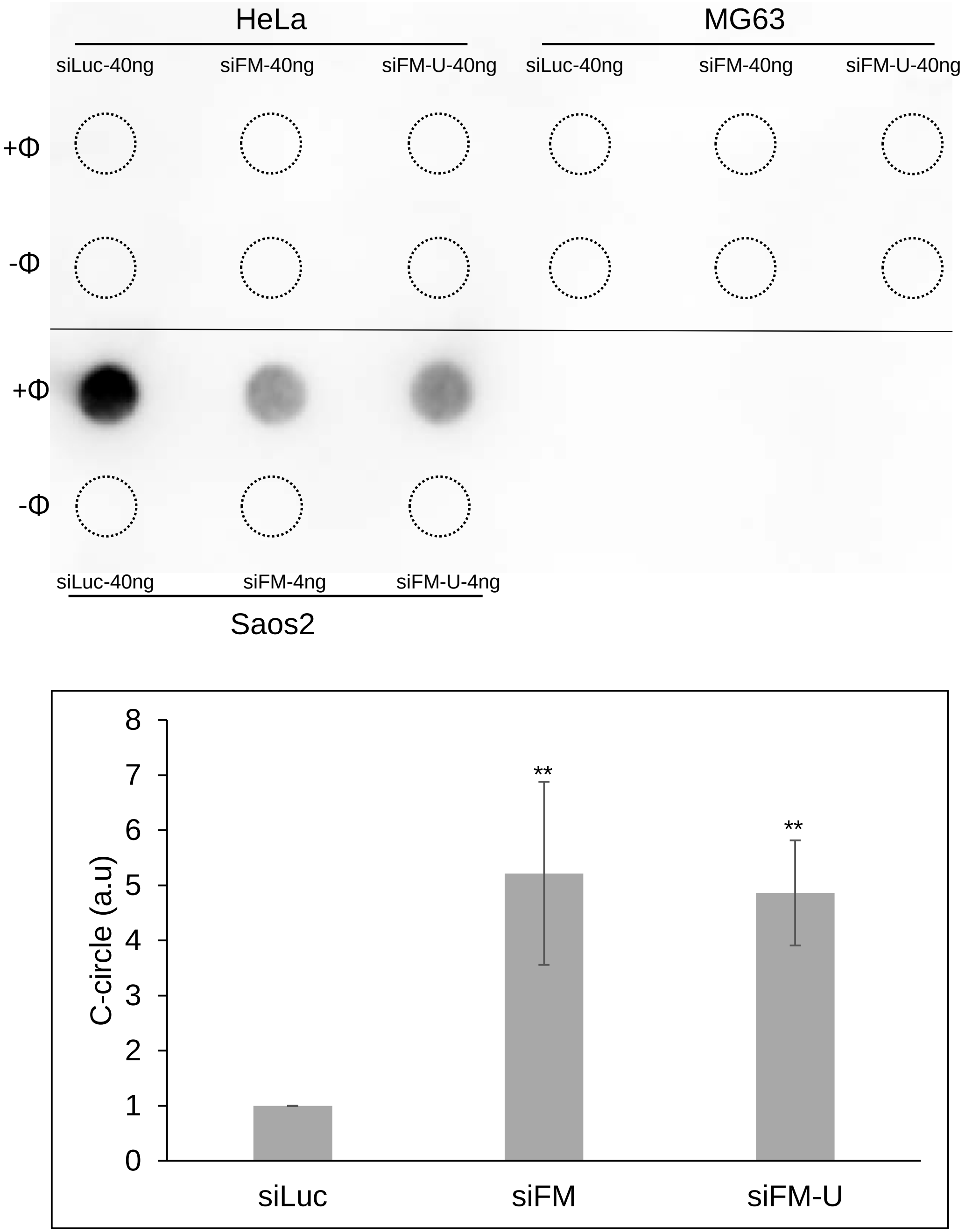
HeLa
MG63
siLuc-40ng
siFM-40ng
siFM-U-40ng
siLuc-40ng
siFM-40ng
siFM-U-40ng
+Φ
-Φ
+Φ
-Φ
siLuc-40ng
siFM-4ng
siFM-U-4ng
Saos2
C-circle (a.u)
0
1
2
3
4
5
6
7
8
**
**
siLuc
siFM
siFM-U

**Fig.S2**

**A**

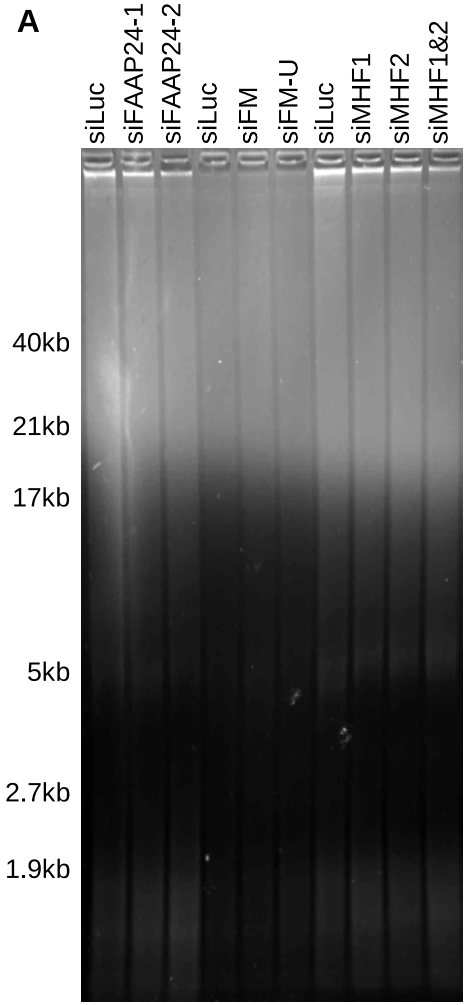


EtBr staining

**B**

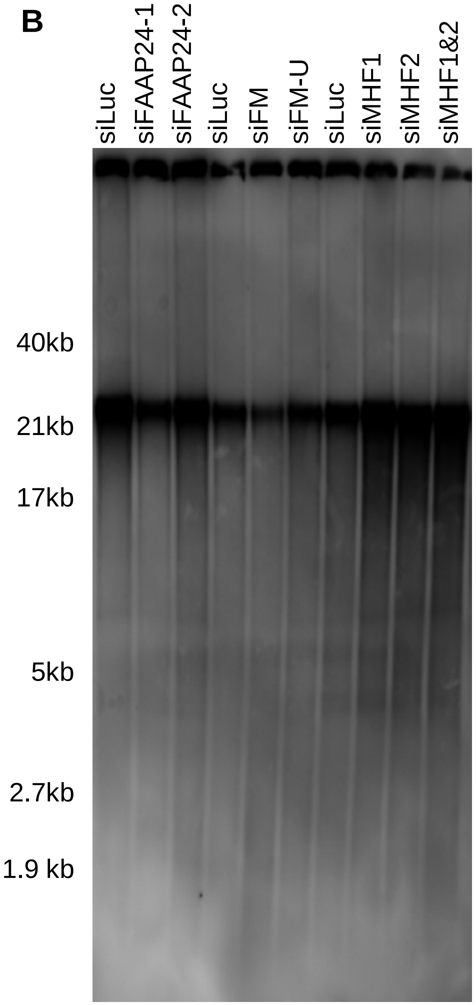


TRF assay

# Fig S3

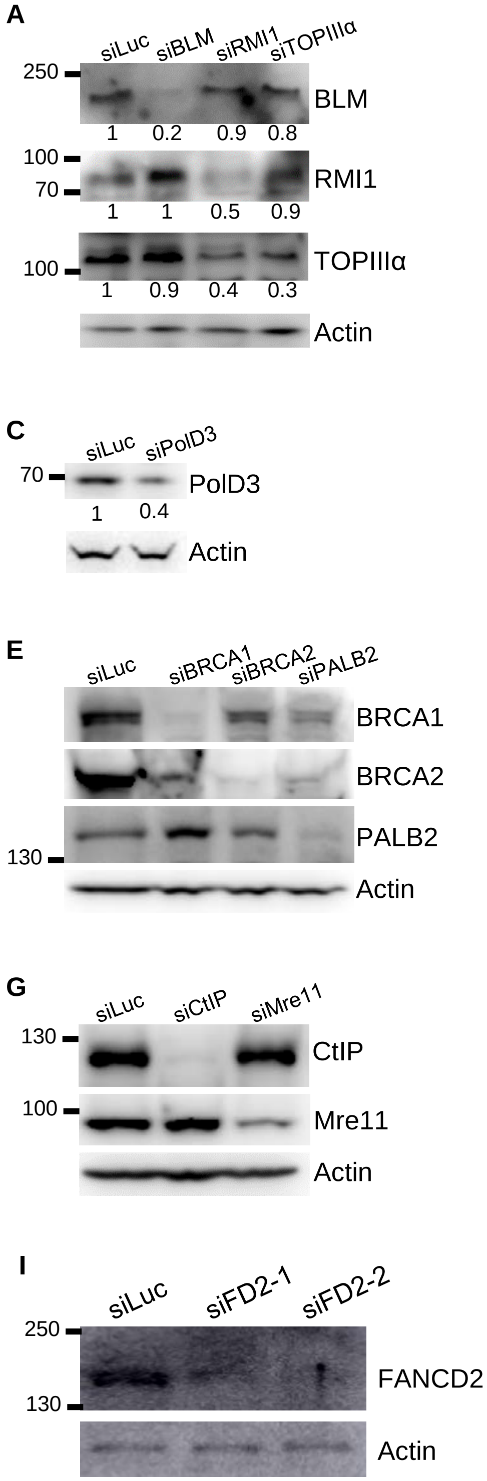

A
siLuc siBLM siRMI1 siTOPIIIα
250
BLM
1 0.2 0.9 0.8
100
70
RMI1
1 1 0.5 0.9
100
TOPIIIα
1 0.9 0.4 0.3
Actin
B
siLuc siATR siChk1
250
ATR
50
Chk1
Actin
C
siLuc siPolD3
70
PolD3
1 0.4
Actin
D
siLuc siPolD1 siPolH
130
PolD1
100
70
PolH
Actin
E
siLuc siBRCA1 siBRCA2 siPALB2
BRCA1
BRCA2
PALB2
130
Actin
F
siLuc siRad51 siRad52
35
Rad51
55
Rad52
Actin
G
siLuc siCtIP siMre11
130
CtIP
100
Mre11
Actin
H
siLuc siSmc5 siSmc6
130
Smc5
1 0.35 0.35
130
Smc6
1 0.52 0.52
Actin
I
siLuc siFD2-1 siFD2-2
250
FANCD2
130
Actin

# Fig.S4

A

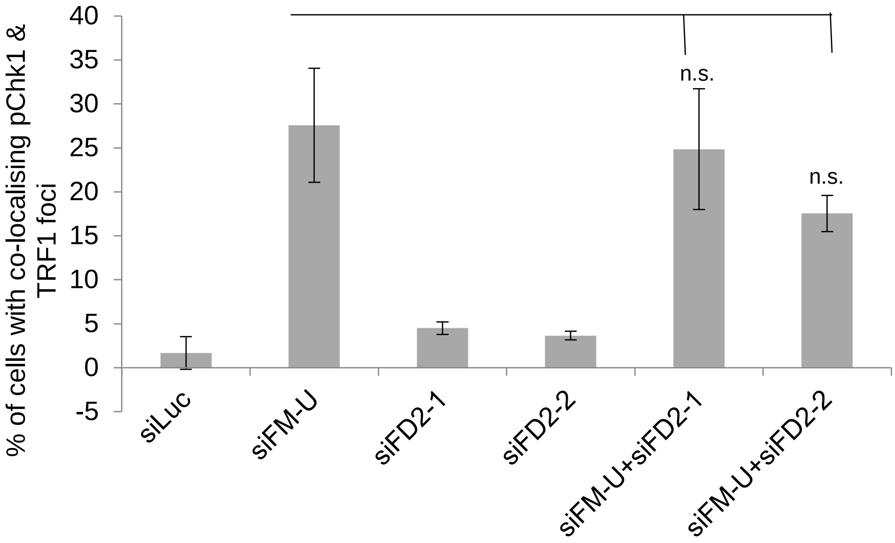

% of cells with co-localising pChk1 & TRF1 foci
40
35
30
25
20
15
10
5
0
-5
n.s.
n.s.
siLuc
siFM-U
siFD2-1
siFD2-2
siFM-U+siFD2-1
siFM-U+siFD2-2


B

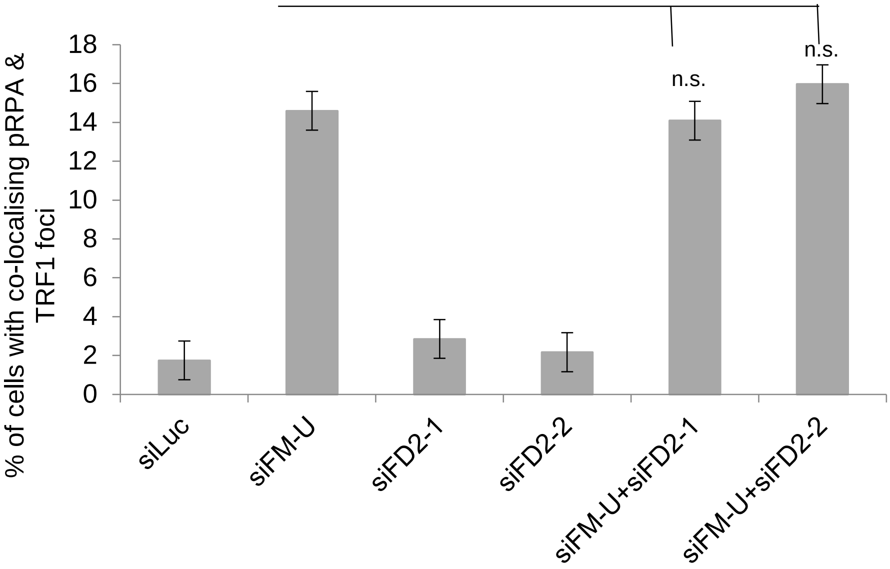

% of cells with co-localising pRPA & TRF1 foci
18
16
14
12
10
8
6
4
2
0
n.s.
n.s.
siLuc
siFM-U
siFD2-1
siFD2-2
siFM-U+siFD2-1
siFM-U+siFD2-2

# Fig. S5

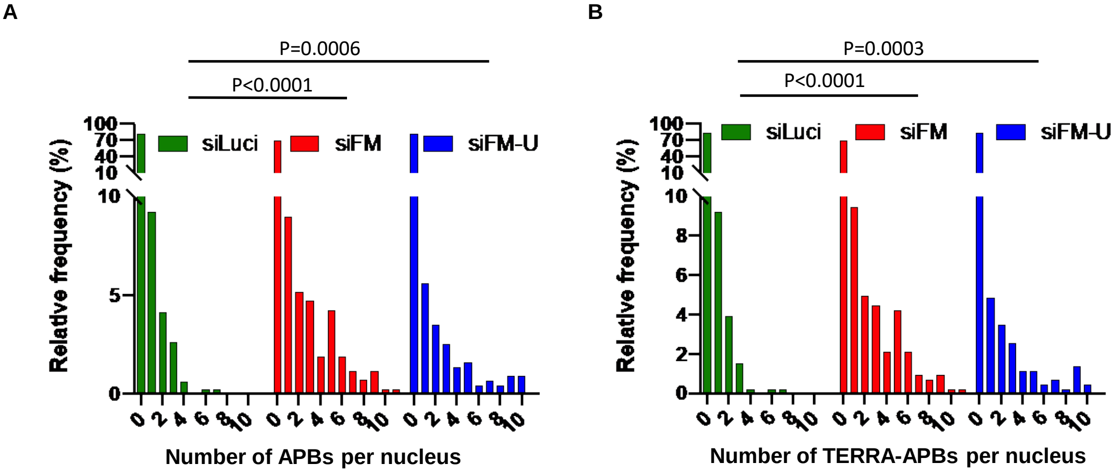
A
P=0.0006
P<0.0001
siLuci
siFM
siFM-U
Relative frequency (%)
Number of APBs per nucleus
B
P=0.0003
P<0.0001
Number of TERRA-APBs per nucleus

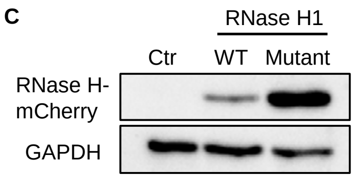
C
RNase H1
Ctr
WT
Mutant
RNase H-mCherry
GAPDH

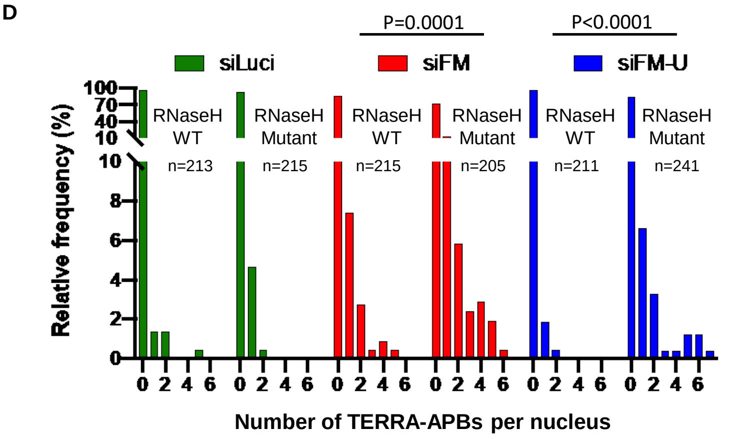
D
P=0.0001
P<0.0001
siLuci
siFM
siFM-U
RNaseH WT
RNaseH Mutant
n=213
n=215
n=215
n=205
n=211
n=241
Relative frequency (%)
Number of TERRA-APBs per nucleus

# Fig. S6

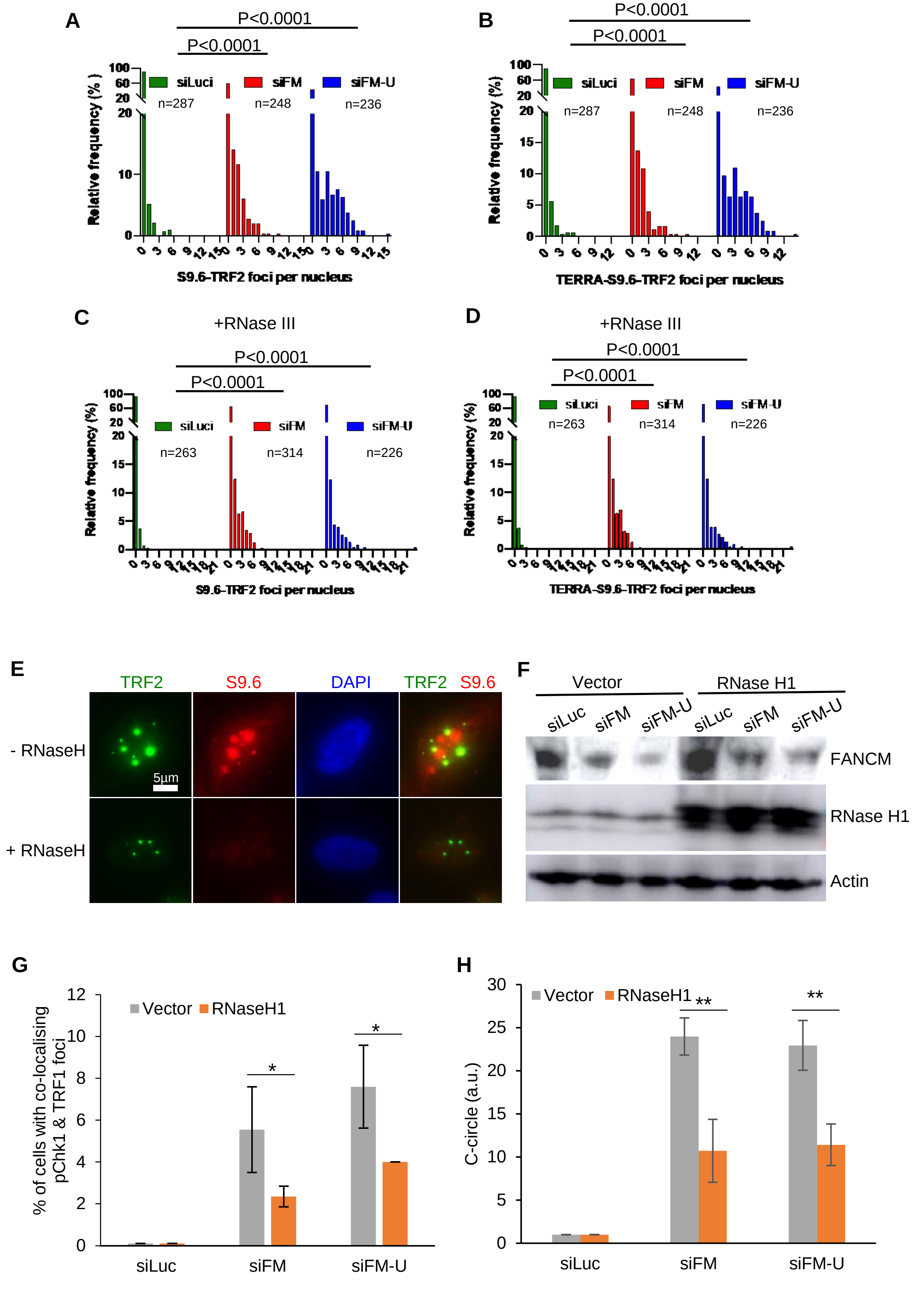
A
P<0.0001
P<0.0001
Relative frequency (%)
siLuci
siFM
siFM-U
n=287
n=248
n=236
S9.6-TRF2 foci per nucleus
B
TERRA-S9.6-TRF2 foci per nucleus
C
+RNase III
n=263
n=314
n=226
D
E
TRF2
S9.6
DAPI
- RNaseH
+ RNaseH
5µm
F
Vector
RNase H1
siLuc
siFM
siFM-U
FANCM
RNase H1
Actin
G
% of cells with co-localising pChk1 & TRF1 foci
Vector
RNaseH1
H
C-circle (a.u.)

Fig. S7

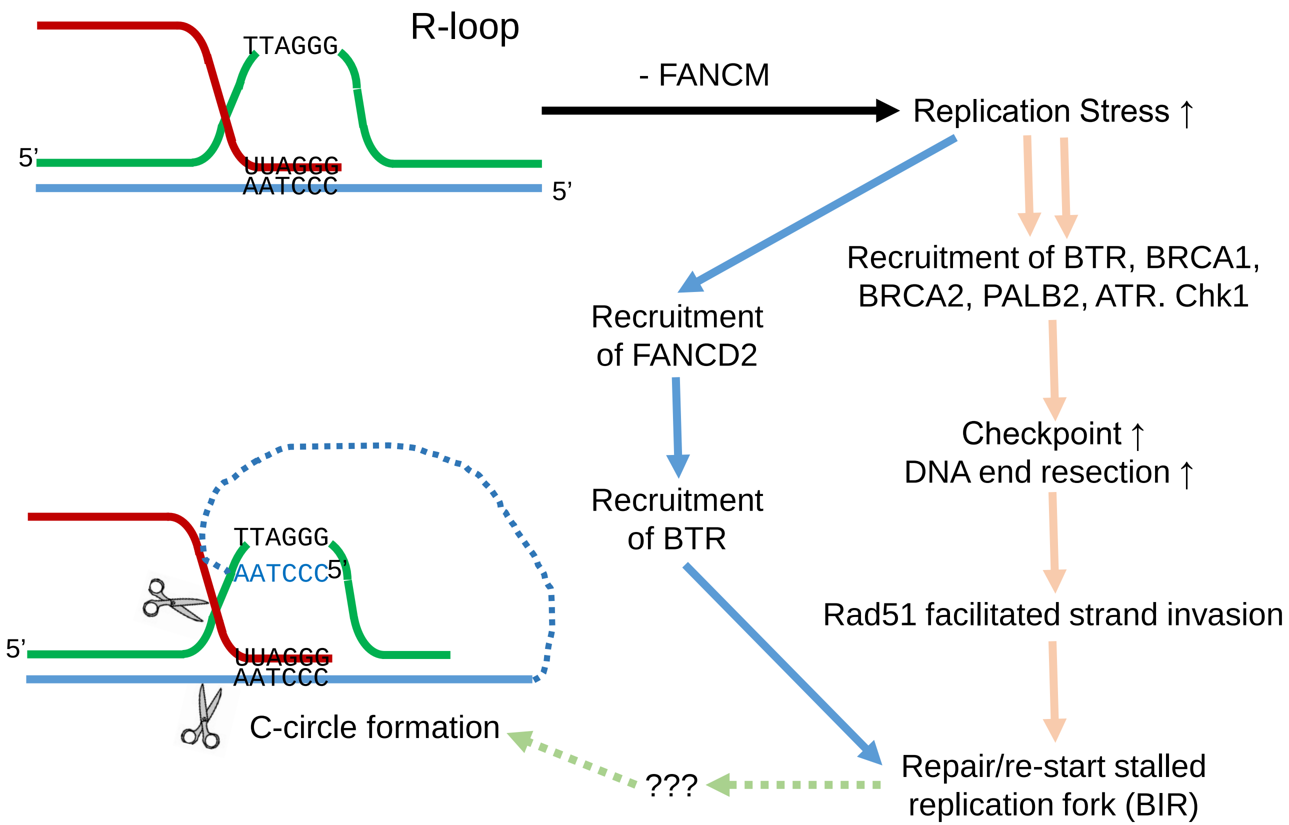

R-loop
TTAGGG
5'
UUAGGG
AATCCC
5'
- FANCM
Replication Stress ↑
Recruitment of BTR, BRCA1, BRCA2, PALB2, ATR. Chk1
Recruitment of FANCD2
Checkpoint ↑
DNA end resection ↑
Recruitment of BTR
Rad51 facilitated strand invasion
TTAGGG
AATCCC5'
5'
UUAGGG
AATCCC
C-circle formation
???
Repair/re-start stalled replication fork (BIR)
